# Development of a personal health management system integrating digital twin technology and smart wearables in China

**DOI:** 10.3389/fpubh.2025.1674111

**Published:** 2025-10-28

**Authors:** Xingyu Wang, Dongfang Yang, Sijia Yu, Yan Zhuang

**Affiliations:** ^1^School of Art and Design, Wuhan University of Technology, Wuhan, China; ^2^School of Art and Design, Tianjin University of Technology, Tianjin, China

**Keywords:** smart wearables, healthy digital twin, personal health management, proactive health, health visualization

## Abstract

**Introduction:**

Traditional health management primarily focuses on treating existing diseases, while neglecting early intervention, prevention, and proactive health maintenance. In contrast, modern Personal Health Management (PHM) emphasizes on prevention alongside treatment. However, the effectiveness of PHM is constrained by several challenges, including limited awareness of proactive health management, the complexity of physiological data generated by smart wearables, and the high cost and scarcity of high-quality medical resources.

**Methods:**

To address these issues, this study explores the integration of smart wearables and digital twin technology in PHM. In the first phase, the study conducted semi-structured interviews with users to confirm current challenges and, with experts to explore promising opportunities in PHM. Drawing on the insights, the paper proposed assumptions regarding the synergistic potential of digital twin technology and smart wearables. In the second phase, the study developed the smart wearables empowered Healthy Digital Twin (HDT) generation model through the incorporation insights and experiences from multiple case studies.

**Results:**

The HDT model comprises four key layers: data monitoring, data aggregation, modeling and computation, and human-computer interaction. In the third phase, the study organized an expert workshop, leading to the development of a comprehensive Product-Service System for full-cycle PHM, based on the HDT model. This system encompasses three main components: a health visualization and early warning, a remote collaborative diagnosis and treatment, and an intelligent recommendation.

**Discussion:**

This system helps to enhance health awareness, optimize daily health management, improve the telemedicine process, and fully utilize the potential of digital healthcare.

## 1 Introduction

There have been significant technological breakthroughs in the fields of Digital Twins (DT) and wearable products, which have shown great potential in Personal Health Management (PHM). Smart wearables have become important tools for daily health management through biosensing technologies (1). Their value in personal health evaluation, early warning, and intervention—such as heart rate monitoring—has been well-acknowledged, as demonstrated by the widespread use of health-monitoring watches. The integration of smart wearables into health management is gradually shifting the focus of healthcare from treatment to prevention. However, the complex health data displayed on smart wearables often fail to provide actionable insights, while contributing to increasing health-related anxiety among users (2).

Meanwhile, DT, also referred to as “information mirroring models,” ensures synchronization between the physical and virtual worlds at an appropriate rate (3). It enhances the visualization of diagnosis, prediction, and treatment, thereby opening new opportunities for PHM. Nonetheless, current DT technology has yet to penetrate the field of PHM deeply. Its potential to provide a comprehensive, secure, and universally connected full life cycle PHM service remains to be realized (4).

From the perspective of health management, there has been a shift in focus from passive diagnosis and treatment to active health maintenance. This paradigm shift has advanced healthcare into the stage of “full life-cycle management,” emphasizing active individual participation across all stages, from disease prevention, diagnosis to treatment and recovery. However, most individuals still lack awareness of proactive health maintenance (5). Non-communicable chronic diseases (NCDs) now constitute the greatest disease burden globally, with cardiovascular diseases, chronic respiratory diseases, diabetes, and cancer being the most prevalent (6). Factors such as poor dietary habits, unhealthy lifestyle behaviors, limited awareness, and failure to monitor one’s health in a timely manner significantly increase the mortality rates associated with NCDs (7).

In light of all these insights, the study focuses on the development of a proof of concept aimed at enhancing the health experience of smart wearable device users. It first employed DT technology to propose a health-oriented design solution – the Health Digital Twin (HDT) model. Building on this, and through the incorporation of design thinking, the study further leveraged the advantages of the HDT model to optimize the full life-cycle PHM service system. The specific research methods and content are elaborated in detail in the following sections.

## 2 Literature review

### 2.1 Lack of health literacy in proactive PHM

Proactive PHM remains a major public health challenge. Approximately 50% of patients with hypertension fail to take medication as prescribed. Many patients lack awareness of the importance of long-term health management, making it difficult to integrate pharmacological treatment and healthy behaviors into daily life (8).

There is close relationship between health literacy and health outcomes. Inadequate health literacy is associated with lower medication adherence, higher rates of adverse clinical outcomes, including complications and mortality, poorer disease management and prognosis among patients with chronic conditions, and increased healthcare costs (9).

### 2.2 Difficulty in understanding health indicators

Difficulty in interpreting health indicators is a significant barrier to the application of remote measurement technologies (RMT). Although RMT can collect large volumes of health data, these data are often presented in complex or highly specialized formats, making it difficult for patients to understand their actual health implications. For patients with depression, epilepsy, and multiple sclerosis, poorly designed or cognitively misaligned data visualizations may cause confusion. Instead of supporting self-management, such indicators can hinder patient engagement and diminish the clinical value that data feedback is expected to provide (10, 11). For example, in pain management, patients often invest considerable effort in collecting data, yet these rich datasets are rarely returned to them in meaningful ways. This suggests that without intuitive visualization, health indicators cannot provide patients with insights, thereby missing opportunities to trigger behavioral change and support treatment (12). In the management of hypertension, while most patients monitor blood pressure at home, only half share these data with their physicians. When home blood pressure data are received, 88% of physicians record the average values as text in clinical notes. Without intuitive visualization, patients find it difficult to understand their health status, and physicians face challenges in integrating these valuable data into clinical decision-making (13). Research has shown that among 203 participants, 136 (67.0%) were confused by technical terms such as medical terminology, reference ranges, the meaning of laboratory values, and their implications for healthcare. Others required support with more complex issues, such as interpreting laboratory results in relation to their medical history (9).

### 2.3 Shortage of healthcare resources

Although the global health workforce is projected to increase from 65.1 million in 2020 to 84.0 million by 2030, a shortfall of 10 million health workers is still anticipated (14). The distribution of these resources is highly uneven, with a 6.5-fold difference in density between high-income and low-income countries, such as the difference between Africa and the Eastern Mediterranean region in the United States. The shortage of critical care physicians and pulmonologists is expected to worsen as the population ages. This imbalance is largely driven by the stagnation in workforce supply alongside the rapidly increasing disease burden associated with aging (15). Recent research further highlights the likelihood of a significant physician shortage (16). For instance, in China, only about 47.3% of middle-aged and older adults have undergone at least one health checkup (17).

### 2.4 Potential role of digital technologies in the PHM process

Digital technologies have the potential to span the entire continuum of health management, from prevention and monitoring to diagnosis, treatment, and rehabilitation. In the prevention stage, wearable devices and health applications can be used to enable real-time monitoring of physiological indicators, allowing users to track their health status and adjust lifestyle behaviors accordingly (18). During monitoring and diagnosis, big data analytics and Artificial Intelligence (AI) can rapidly identify health risks, support accurate diagnosis, and improve clinical efficiency. DT technologies further create personalized virtual health visualization that simulate and predict health conditions dynamically, offering deeper data support for precision medicine (19).

In treatment, telemedicine can provide patients with intervention plans, reducing both the cost and time of care (20). In the rehabilitation stage, digital technologies can support long-term health management and reduce recurrence risks through health record management and follow-up systems (21). Overall, digital technologies can not only enhance the efficiency and accuracy of PHM but also promote more effective allocation of medical resources and foster greater public health awareness (22). Recent advances in informatics can further enable the effective collection and detailed analysis of human health data. The development of personalized healthcare DT makes it possible to simulate individual health trajectories and disease progression. By incorporating diverse health indicators, DTs allow quantitative analysis of life processes, provide dynamic health guidance, and optimize treatment strategies. This innovative approach can strengthen the mathematical foundations of biological mechanisms, reshape existing clinical practices, and advance truly personalized medical care (23).

Although digital technologies have such great potential, current research has not yet proposed a system capable of employing these functions, to support whole life-cycle PHM. In other words, there remains a need for a PHM System that integrates these functions to address the identified challenges. In summary, this research focuses on three primary challenges: the general lack of awareness in proactive PHM, the difficulties users face in interpreting daily health indicators, and the shortage of healthcare resources. Accordingly, the guiding research question is: How can digital technologies be employed to enable effective and efficient PHM, thereby addressing the challenges of limited awareness, difficulties in understanding indicators, and insufficient medical resources?

## 3 Materials and methods

Addressing the complex challenges surrounding public health demands a multidisciplinary approach that integrates insights from various disciplines (24). To tackle these challenges, this research built interdisciplinary collaboration among designers, healthcare givers, and digital technology engineers. The objective was to develop a full-cycle PHM system by integrating knowledge from different fields.

The study employed a mixed methods approach, adopting design thinking with the process of empathize – define – ideate – prototype – test. During the empathize and define phases (phase 1 and phase 2), the study examined the key dilemmas and opportunities within PHM. As a result, the research developed the HDT model by integrating functions of DT and smart wearables.

The ideation, prototyping and testing were conducted and finished during a workshop (phase 3). Finally, the research proposed a PHM system aimed at enhancing individuals’ awareness, capability, and access to proactive healthcare.

### 3.1 Phase 1: identifying user needs and research opportunities - interviews

This phase involved interviews with users and experts to identify challenges in PHM and opportunities for digital technology intervention. It began with semi-structured interviews with 40 participants, selected through simple random sampling. The study targeted urban residents in Wuhan and Nanjing who had expressed willingness to participate. The aim was to uncover dilemmas and challenges in PHM, and to provide direction for the study. Among the participants, 21 were male and 19 were female; 24 were young adults aged 20 to 35, 12 were middle-aged adults aged 36 to 59, and 4 were older adults aged 60 and above. The selection of interview themes was informed by product–service system design and user experience design theories, focusing on three areas: fostering proactive health awareness, usability of wearable devices, and health management experience. The research questions were refined through repeated discussion among the four authors and reviewed by a domain expert. During the interviews, the moderator took notes and made audio recordings. Afterwards, the recordings were transcribed, and two researchers independently coded the transcripts according to the three themes. This process ultimately distilled and validated the three major pain points and challenges identified in the literature review. The key findings on health management derived from the interviews are detailed in the results section. All participants gave informed consent to participate in the study. The study was reviewed and approved by the Academic Committee of our institution, and ethical clearance was obtained.

In addition, we conducted in-depth interviews with 8 experts from the fields of health management and digital healthcare. These interviews explored emerging opportunities for PHM in the digital era, such as the feasibility of the DT and the mechanisms through which smart products can effectively support PHM.

### 3.2 Phase 2: developing a framework for the HDT generation model - case study

The method employed in this phase was case analysis. By systematically deconstructing, interpreting, and synthesizing specific health management cases, the study aimed to extract design principles, draw lessons, and identify applicable practices for constructing a smart wearable- empowered HDT generation model from the perspective of user-centered health management. Given the limited number of existing studies on this topic, a multiple-case study approach was adopted (25, 26). The case selection criteria focused on initiatives that leverage digital and intelligent technologies to enhance the visualization of personal health information and provide potential disease warnings. Two cases offered detailed information from various sources, including websites, documents, or analyses conducted by other researchers. By examining the primary concerns, operational processes, and data requirements of each case, this study established the HDT generation model.

### 3.3 Phase 3: development of a full-cycle PHM system –workshop

The PHM system was then developed through a process of ideation, prototyping and testing. A workshop group was assembled, including designers, users, smart wearable manufacturers, healthcare professionals, community workers, and caregivers. Participant selection followed stakeholder identification principles in service design, distinguishing between core and secondary stakeholders. Core stakeholders included users, wearable device manufacturers, and healthcare professionals, while community workers and caregivers were considered as secondary stakeholders. The five-hour workshop employed brainstorming and co-design discussions to explore and refine a PHM system design. During the design discussions, the designer acted as the project facilitator, elucidating the relationships and interactions among various stakeholders within the PHM system. Based on the outcomes of the brainstorming, a PHM Product-Service System (PSS) was proposed for full life-cycle health management that links the HDT. At last, we presented the PHM-PSS to all experts involved in the workshop and get their feedbacks for further refinement.

**Figure 1:**
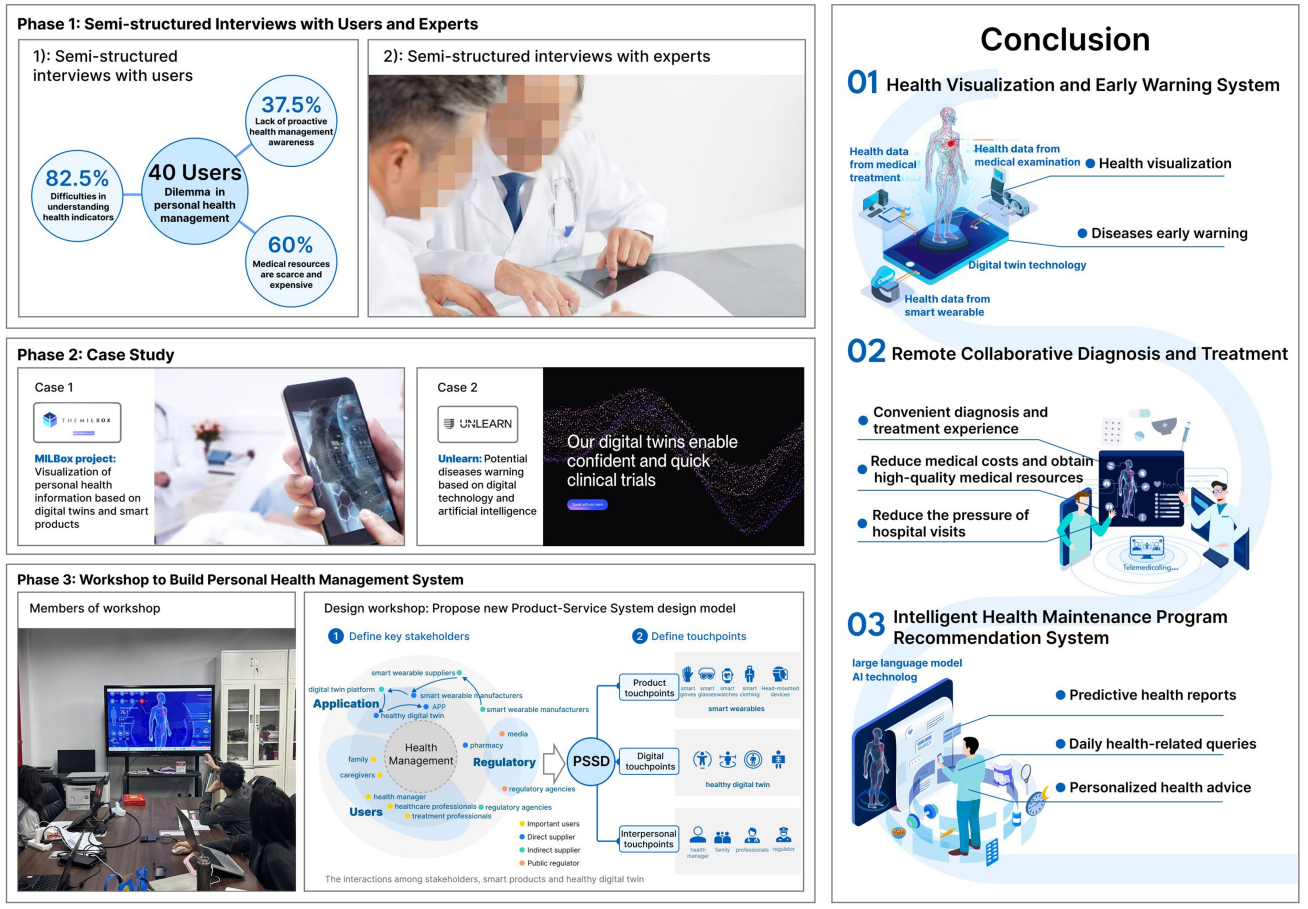
The research methodology.

The methodology of this study is illustrated in Figure 1.

## 4 Results and findings

Following the design thinking approach, during the empathize stage, we identified three main challenges in PHM. Building on these, we defined initial opportunities through expert consultation and case studies analysis in the define stage. Synthesizing these findings, the study proposed the PHM-HDT generation model. Subsequently, through an iterative process of ideation, prototyping, and testing, the study developed a full-cycle PHM-PSS.

### 4.1 Construction of PHM-HDT generation model

The key issues within PHM can be categorized into three main areas. First, 37.5% of users did not use smart wearables, reporting that the long intervals between physical examinations hindered the timely identification of potential health risks. Beyond conditions detected during routine check-ups, many overlooked early warning signals from their bodies, often missing the optimal treatment window by the time they sought medical attention. Second, 82.5% of smart wearable users indicated that daily physiological indicators—such as blood glucose, heart rate, and electrocardiogram readings—were difficult to interpret, which reduced their motivation to engage in health management. This finding aligns with previous research: despite the widespread adoption of fitness trackers and smartwatches, users frequently struggle to make sense of physiological data that are typically presented in technical medical terms (47). The gap between the “encoding” of these metrics and their “decoding” by users further complicated comprehension (27). Moreover, the complexity of disease mechanisms and the general lack of professional health management knowledge among individuals constrained effective PHM (48). Third, while 60% of users acknowledged the importance of early intervention, traditional face-to-face medical consultations remained time-consuming, leading to delays in seeking care. In summary, the central challenges to be addressed by this study are: limited proactive awareness of health management, difficulties in interpreting daily health indicators, and insufficient access to healthcare resources.

To address these challenges, 3 experts in digital care suggested that smart wearables and Digital Twin (DT) technology could provide viable solutions. DT represents a digital replica of a physical entity—whether an object, system, process, or even a city—constructed within a virtual environment. Leveraging this approach, data from smart wearables can be transformed into a DT of human body, with information visualization methods enhancing the readability and interpretability of health data. The other 5 experts emphasized that health information visualization can present vital signs and health maintenance solutions in more accessible ways, thereby improving users’ experience of proactive PHM.

#### 4.1.1 Case study

##### 4.1.1.1 1st case_MILBox project

###### 4.1.1.1.1 Case information

To establish the PHM-HDT model, this study first analyzed the MILBox project, which leverages DT to visualize personal health information. The project is led by the Media and Innovation Lab (MIL) at the University of Miami Miller School of Medicine, in collaboration with Amazon Web Services (AWS) and the Open Health Network. MILBox aims to create a HDT by integrating personal data and medical records collected from hospitals and physical examination centers, presenting this information in a visualized format.

###### 4.1.1.1.2 Insights from the case

The process of constructing a HDT in the MILBox project involves the following stages: health data collection, transmission, analysis, generation, and visualization. Two critical prerequisites for incorporating DT into health management were identified: (1) the availability of comprehensive personal health data as the foundational input (24) and (2) the support of a DT platform to present the HDT (28). This case study highlights three essential data layers necessary for developing a DT model: the Health Data Collection Layer, the Modeling and Calculation Layer, and the Human-Computer Interaction Layer.

However, the MILBox project focuses on the medical institution scenario rather than PHM scenario. It does not comprehensively address how to enhance the overall health management experience or encourage users to engage proactively with their health. Building on these insights, this study refined the health data collection layer for PHM by expanding data sources to include vital sign monitoring data from smart wearables. In doing so, the study enlarged the scope and accessibility of health data, paving the way for a more comprehensive and user-oriented PHM experience.

##### 4.1.1.2 2nd case_Unlearn

###### 4.1.1.2.1 Case information

From a feasibility perspective, several research institutions have begun employing AI technologies for early warning of specific diseases. This progress offers strong technical support for the development of health products with predictive functions. For example, Unlearn, a US-based company, uses user-authorized health data from multiple sources to construct an HDT and integrates AI to support proactive disease prevention (29). Specifically, Unlearn integrates user’s cognitive scores with the Alzheimer’s Disease Assessment Scale to predict, up to 18 months in advance, the likelihood of getting Alzheimer’s disease. Although current technical constraints prevent complete accuracy in disease risk predictions, empirical evidence indicates that Unlearn’s AI algorithms reach an accuracy rate of 84% in predicting dementia-related conditions (29).

###### 4.1.1.2.2 Insights from the case

This case underscores the potential of HDT to provide early warnings of potential health risks. Similar to the first case, the data utilized in this example primarily derive from personal health records and physical examination results, without incorporating real-time physiological metrics from daily life. This limitation restricts the application of HDT to post-examination contexts and prevents it from dynamically reflecting changes in an individual’s health status.

##### 4.1.1.3 Insights from both cases

To developed the PHM-HDT generation model within our study, smart wearables play a pivotal role by continuously monitoring user’s daily health metrics (30). It is essential to incorporate real time health data collected from individuals’ daily activities (4). These data, along with medical records and physical examination results, can be aggregated via cloud based platforms (31). As a result, we propose integrating smart wearables with DTs, refining the health data collection layer (identified in Case 1) into a data monitoring layer and a data aggregation layer to address the limitations of health data sources in daily health maintenance. With user authorization, smart wearables serve as the critical medium connecting individuals to the DT generation platform and to broader medical resources, thereby enabling dynamic and comprehensive health visualization and management in everyday contexts. Our model can further integrate early warning functions into daily health maintenance scenarios to enhance proactive health management and facilitate timely interventions (insights from case 2).

#### 4.1.2 The structure of the PHM-HDT generation model

**Figure 2:**
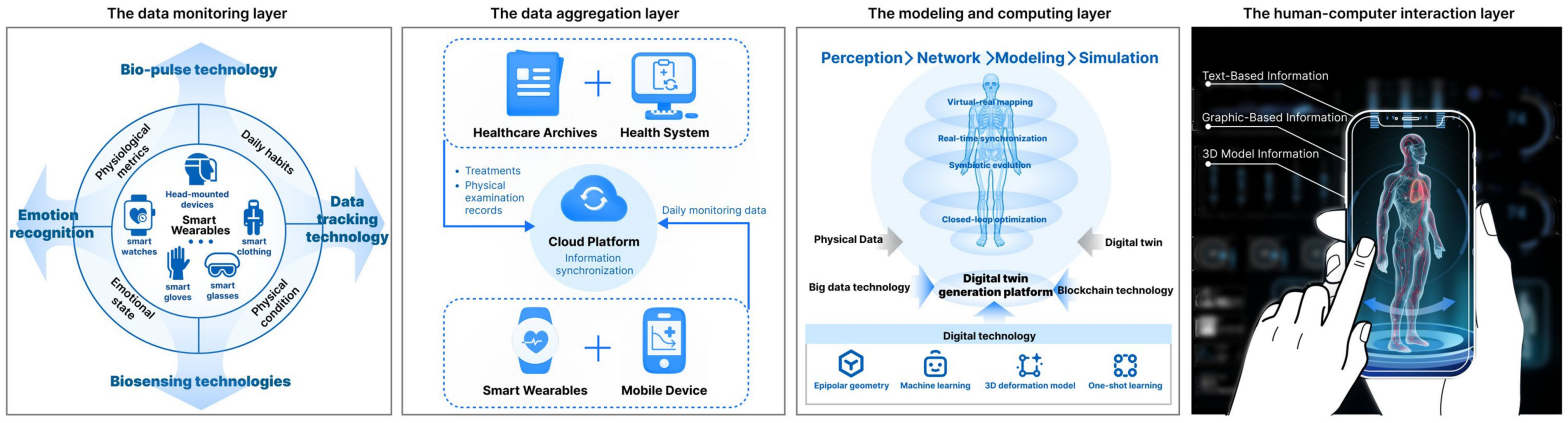
The HDT generation model.

This study constructs a smart wearable empowered PHM-HDT generation model that dynamically and accurately reflects the user’s health status in real time. The model typically includes four key layers: a data monitoring layer, a data aggregation layer, a modeling and computation layer, and a human-computer interaction layer (see Figure 2). This encompasses three essential components: the physical model, the generation platform, & the virtual model (32, 46). Consistency between the DT and the physical counterpart is critical, and data collection, transmission, and integration form the necessary foundation (33, 34).

The data monitoring layer. This layer focuses on collecting comprehensive personal physiological data, to provide foundational support for the construction of the HDT. Smart wearables capture users’ daily physiological metrics, such as blood pressure, electrocardiogram (ECG), blood oxygen levels, and heart rate, in real time using bio-pulse technology (35). Additionally, they record daily habits such as exercise routines and sleep patterns. It is important to emphasize that the collection and application of this daily physiological data must be conducted with explicit user authorization, ensuring user privacy and consent throughout the process (36).

The data aggregation layer. It integrates daily physiological data collected by smart wearable devices with personal health records, such as physical examination records, medical diagnoses, treatments, and medical histories from healthcare archives. For personal health records, we suggest that after undergoing a physical examination or receiving medical treatment, doctors create individual electronic health records on a secure cloud platform using blockchain technology. Together, these sources form the complete dataset for each individual, resulting in a “one person, one database” structure. To safeguard user privacy, this process incorporates encryption and authorization management for the transmission of health data. Advanced encryption technologies are employed to ensure the strict protection of personal health data, mitigating the risk of data breaches and unauthorized access (37).

The modeling and computing layer. This layer builds upon the inputs from the monitoring and aggregation layers. It constructs a HDT by integrating health data from multiple sources. This process relies on advanced AI techniques, including machine learning, 3D deformation modeling, polar geometry, and one-shot learning, to render an individual’s health status, i.e., the HDT, using a DT generation platform.

The human-computer interaction layer. The information generated by the modeling and computing layer is integrated into the visual design of the HDT. This layer presents the HDT to users in a clear and comprehensible manner, guided by the principles of information visualization. It focuses on designing the visual representation of the HDT from a user experience perspective and presenting it on the user’s digital devices. This aims to provide users with a dynamic, easy-to-read, and trustworthy depiction of their health status. From the perspective of information visualization design, the interactive interface of the HDT incorporates textual data, graphical elements, and 3D model representations.

### 4.2 Development of a full cycle personal health management product-service system (PHM-PSS)

The PHM-PSS encompasses three touchpoint categories: product touchpoints, digital touchpoints, and interpersonal touchpoints. For PHM, smart wearable devices function as the product touchpoints connecting users to the HDT generation platform. The HDT itself serves as a pivotal digital touchpoint within the PHM-PSS. Additionally, interpersonal touchpoints are represented by doctors, health managers, family members, regulatory authorities, and other stakeholders supporting users’ health management.

Through the initial phase of this study, challenges in PHM were identified, which can be categorized into: over-specialization of health information, delays in daily disease warnings, a limited health management approach, an imbalance between medical service supply and demand, and insufficient utilization of intelligent technologies. To address these issues, this paper proposes leveraging smart wearable devices and DT technology to build an HDT. It is important to note that limiting connectivity to specific brands of wearable devices can lead to severe data silos. To avoid this, the proposed system design is based on the TwinRCTs DT generation platform or the PatientSphere 2.0 technology platform, both of which adopt open API interfaces to support multi-brand and multi-type devices. This approach effectively breaks down data barriers. Manufacturers of wearable devices can develop synchronization programs according to the platform’s interface standards, ensuring compatibility between devices and the platform. Through the user’s wearable devices or smartphone, the HDT serves as an interactive tool for real time monitoring and management of personal health.

Based on literature research and case analysis, the TwinRCTs DT generation platform and the PatientSphere 2.0 technology platform have already demonstrated the capability to effectively support the construction of HDT models. Building on this foundation, the present study explores, from a design perspective, how these platforms can be integrated into the entire process of PHM. Based on insights from expert brainstorming workshops, the study divided the PHM process into three chronological stages: disease prevention, diagnosis and treatment, and rehabilitation. These three stages follow the temporal logic of PHM, focusing, respectively, on maintaining personal health, treating disease conditions, and supporting recovery. By integrating the HDT into these stages as digital touchpoints, this study designs a comprehensive PHM-PSS. The proposed system incorporates the following sub-systems:

- Health Visualization and Early Warning System.
- Remote Collaborative Diagnosis and Treatment System.
- Intelligent Health Maintenance Program Recommendation System.

Together, these components form a holistic full-cycle PHM-PSS, addressing key pain points and optimizing health outcomes.

#### 4.2.1 Design of health visualization and early warning system

The personal health visualization and early warning system is mainly oriented to personal daily life scenarios. Daily health data from smart wearables and medical examination and treatment are uploaded to the DT platform, to construct a HDT with health visualization and disease warning functions. First, the personal health e-archive is uploaded on the cloud platform through blockchain technology, covering personal health data. Second, by wearing smart wearable devices, real time monitoring of the user’s main physical characteristics values, and other major physiological values, as well as the individual’s daily exercise habits, sleep status and so on are uploaded on the cloud platform. Finally, all these health data are transmitted to DT generation platforms, such as the AI-powered TwinRCTs and the PatientSphere 2.0, to generate an HDT.

The HDT could help enhance individuals’ awareness of proactive health management (38). On the one hand, the HDT will clearly display the user’s health status and dynamically update with changes in the smart wearable monitoring data. This guides the user to actively pay attention to their own health status to prevent diseases before they occur; on the other hand, the HDT will be used as an early warning system for potential diseases.

The interactive interface of the HDT incorporates 3D models, text, images, and animations to comprehensively visualize an individual’s overall health status, track health status over time, and highlight potential disease risks. The design elements, functional descriptions, and technical support are detailed in Table 1. Textual information primarily supplements key health indicators, emphasizing values that signal potential health issues rather than serving as the primary message (39). Graphical information adopts a design semiotics approach, converting key metrics monitored by smart wearables into a two-dimensional visual format. This includes graphical representations of conventional health parameters such as blood pressure, blood lipids, and blood sugar levels. The 3D model utilizes data from multiple sources to simulate and visualize the detailed condition of an individual’s overall health condition. As a core component of the HDT, the 3D model is pivotal to achieving effective health visualization.

**Table 1:** Interactive interface design of the HDT.

Type of information	Text-Based information	Graphic-Based information	3D Model information
Scenario illustration	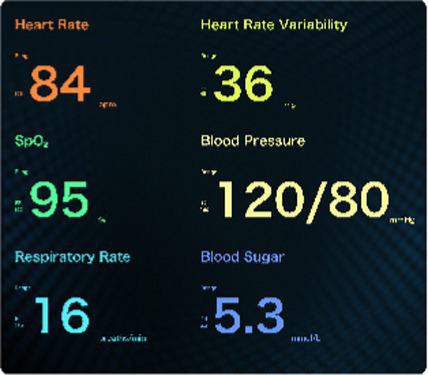	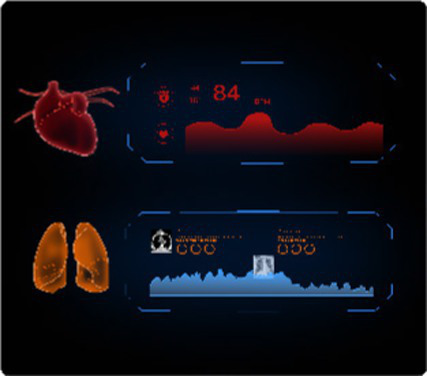	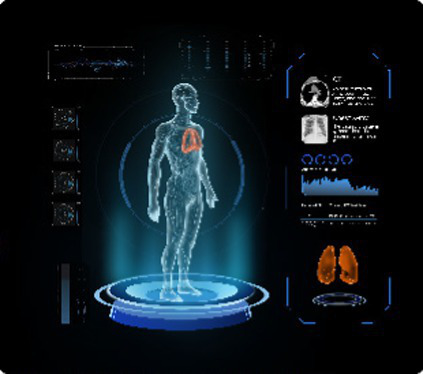
Design elements	Titles: Font size 34–38 px. Body text: 26–30 px. - Spacing: 0.2–0.5x font height. - Font: Highly legible. - Color: Green for normal, red for disease alerts.	The numerical data, such as blood pressure and blood sugar, is translated into visual formats like charts and icons. Symbolic graphics represent different physical indicators. The brightness and purity of colors are adjusted based on the severity of disease warnings. A consistent visual style is maintained across colors, text, and charts to create a unified interface.	3D imaging is utilized to interpret and visualize body health status. When abnormalities are detected, a red warning is highlighted on the affected organ in the 3D model. Enhanced interactivity allows users to navigate the model and access detailed health information, supported by animation effects.
Function illustration	Supplements health information with brief textual descriptions of key health values and sub-health conditions.	A two-dimensional visual interface processes data monitored by smart wearables, providing an intuitive display of routine physical indicators.	The 3D simulations focus on representing major organ diseases.
Technical support	Built-in sensing and cloud management.	AI algorithms and digital imaging.	Digital modeling and Unity3D technology.

**Figure 3:**
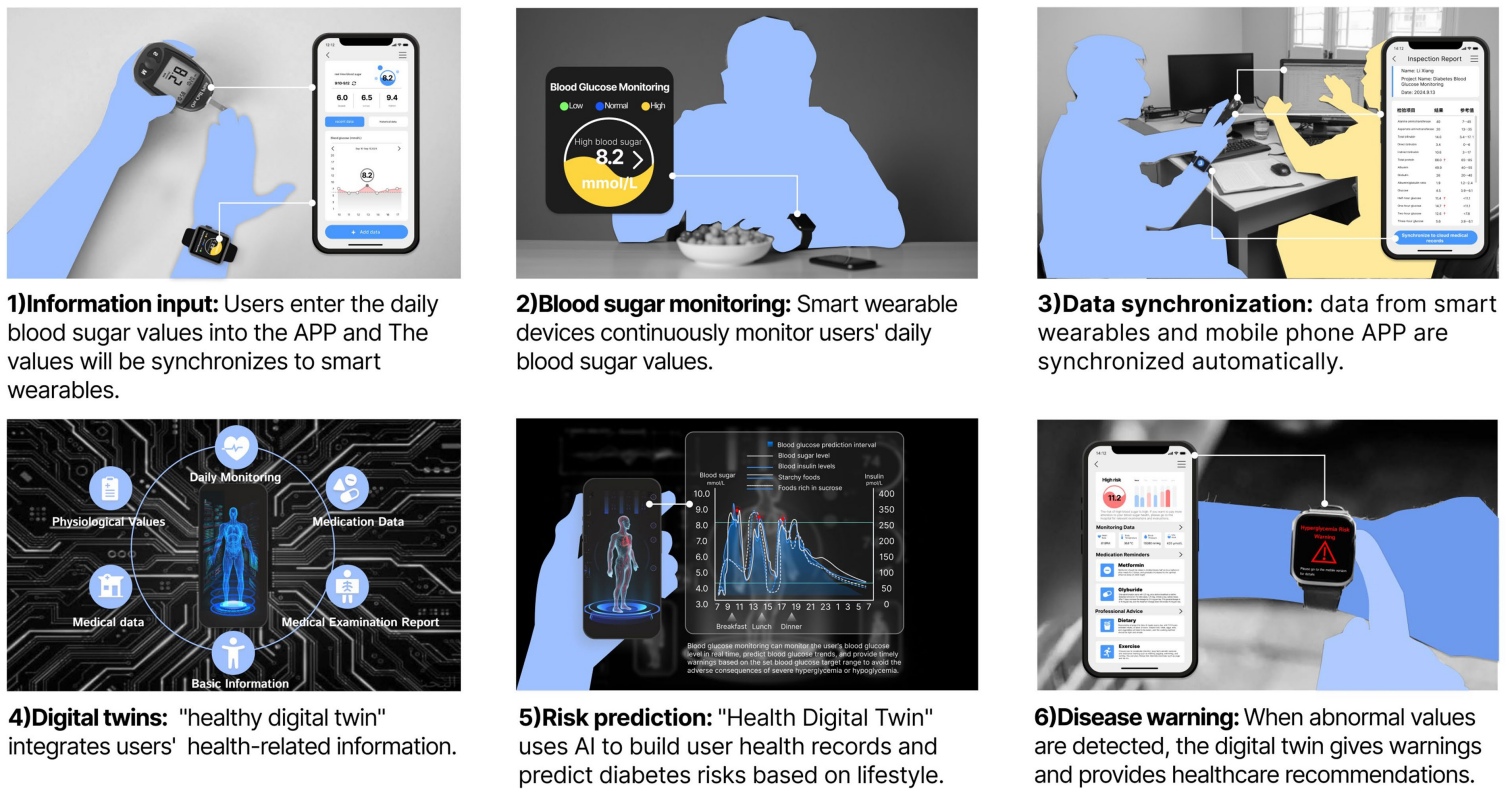
The storyboard of a proactive early warning system for potential health issues enabled by the HDT.

For disease warning, an “unsupervised” deep learning approach is employed to predict an individual’s potential disease risks and confidence intervals using multi-channel health data. For example, Unlearn’s Boltzmann Encoder Adversarial Machine (BEAM) has demonstrated success in accurately predicting the risk of Alzheimer’s disease (29). The accuracy of these predictions improves with the diversity and richness of personal health data from various sources (40). Within the system, this aggregated physiological data is transmitted to a DT generation platform. The platform integrates this data with the BEAM AI system to create a HDT capable of disease risk prediction. The system alerts users to potential health risks associated with their current lifestyle and provides recommendations for early intervention. For instance, the system can periodically monitor an individual’s blood glucose levels through smart wearables while aggregating medical examination results and other physiological data. This information is used to construct a HDT that visualizes periodic blood glucose changes. Combined with the BEAM AI system, the DT generates a personalized health profile, predicts diabetes risk and its likely onset time based on current habits, and raises awareness of necessary health precautions. Figure 3 illustrates the storyboard of a proactive early warning system for potential health issues enabled by the HDT.

#### 4.2.2 Design of a remote collaborative diagnosis and treatment system

Compared with traditional in-person diagnosis and treatment, telemedicine leverages intelligent and digital technologies to deliver significant advantages (41). Online healthcare services can substantially reduce personal travel and time costs associated with accessing healthcare. For regions with limited access to high-quality healthcare resources, as well as for the older adults or individuals with mobility impairments, remote healthcare enables expert doctors to provide consultations and participate in medical discussions from a distance (42). For non-complex and common illnesses, remote healthcare shifts medical services away from hospital settings, offering patients a more convenient and accessible healthcare experience.

One of the primary obstacles to the widespread adoption of telemedicine is the inability of doctors to access patients’ real time physiological data. While, during in-person consultations, doctors can directly assess patients’ conditions through medical tests and imaging examinations. Additionally, some patients, particularly older adults, often struggle to accurately and comprehensively describe their symptoms using online communication tools. An HDT, which mirrors an individual’s routine physiological values and periodic health status, offers a promising solution to this problem in telemedicine. From a health Product-Service System Design perspective, smart wearables serve as the product touchpoints for telemedicine, while the HDT, generated through the integration of wearable devices and DT platforms, functions as the information touchpoint.

**Figure 4:**
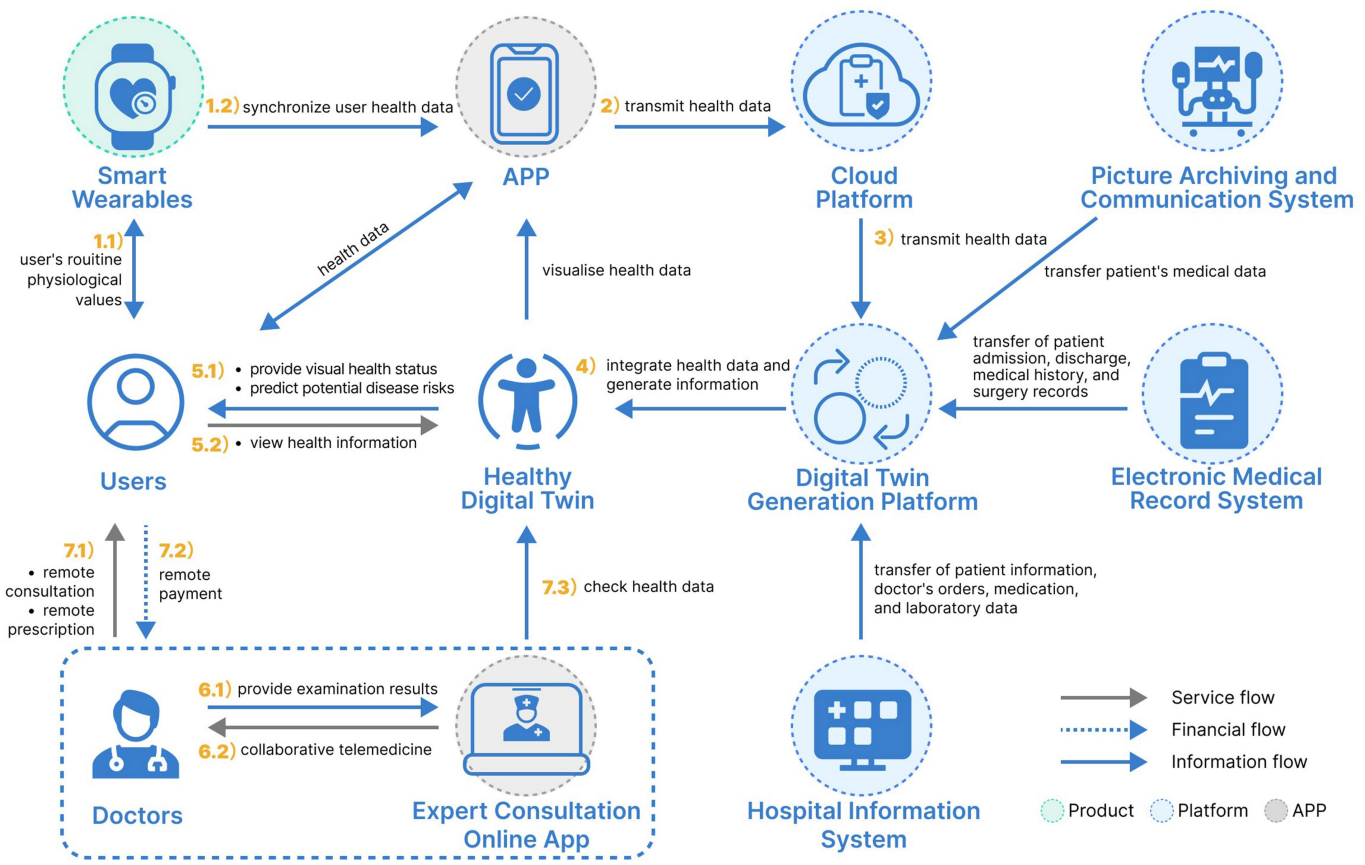
A remote collaborative diagnosis and treatment system design.

Accordingly, the design process of the collaborative telemedicine system based on the HDT is as follows: First, the user’s routine physiological values are monitored through smart wearable devices. At the same time, the user’s health data such as medical visits, medication, and diagnosis and treatment are aggregated to the cloud; Second, a HDT that maps an individual’s physical state is constructed through the AI-powered TwinRCTs DT generation platform or the PatientSphere 2.0 technology platform; Third, if the HDT presents a poor health status, or if a potential risk of disease is predicted, the individual can connect directly with a doctor online for a remote consultation. Fourth, the telemedicine service uses the HDT as an important reference for doctors and patients to communicate about the patient’s condition online. The doctor can view the corresponding DT online and give health maintenance advice based on the patient’s inquiry. Fifth, if an individual’s condition is complex, the doctor can send the user’s DT and other test results online to expert doctors for remote consultation. The above system design is shown in Figure 4. Within the traditional medical model, the doctors directly contact the patient, while the DT reshapes the traditional medical model through remote intelligent diagnosis and treatment services.

**Figure 5:**
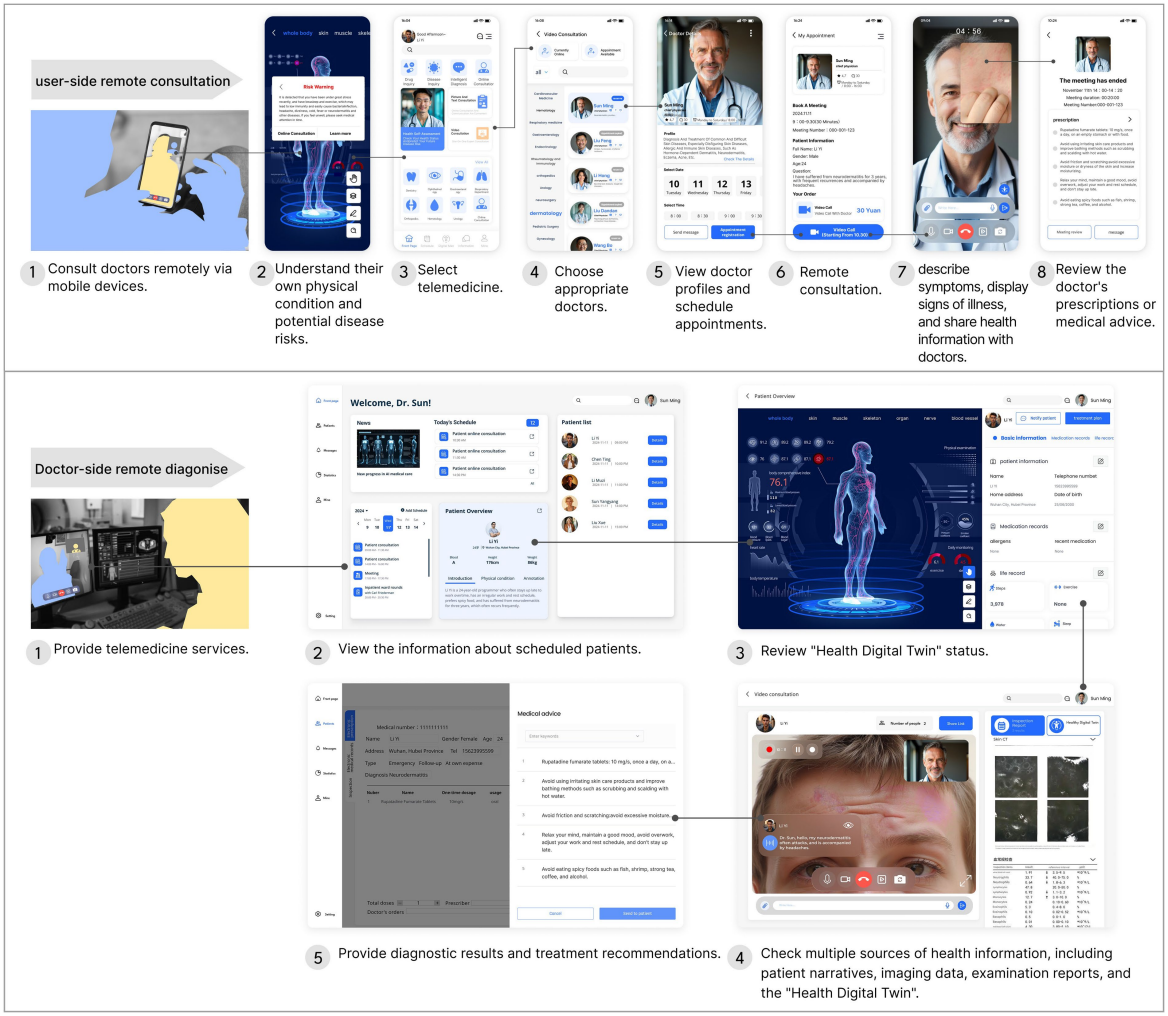
The interactions between the user and the doctor within the telemedicine.

In the remote collaborative diagnosis and treatment system, the interactions between the user and the doctor are shown in Figure 5 below. Users can consult doctors remotely using a mobile device, while doctors provide online diagnosis and treatment services through a computer (43). Within this system, the HDT acts as a crucial medium to facilitate communication between doctors and patients regarding health conditions. This digital representation offers a clear visualization of an individual’s physical state at various stages, enabling doctors to make more precise diagnoses during remote consultations (44). Additionally, for more complex cases, doctors at medical institutions can invite expert physicians to participate in collaborative remote consultations. This approach can alleviate the financial burden on individuals and reduce the need to travel to large hospitals, at the same time, improving access to high-quality medical care.

#### 4.2.3 Design of intelligent recommendation system

With the increasing prevalence of wearables such as smartwatches and fitness trackers, their combination with the DT technology offers a clear and intuitive representation of an individual’s health status. However, if the DT reflects a suboptimal health state or minor illnesses, some users may experience heightened health anxiety. Thus, it is essential to develop strategies to mitigate user anxiety when encountering health information. Currently, Google researchers have refined a large language model specialized in personal health, PH-LLM, based on the Gemini model (45). By integrating health data with PH-LLM, users can promptly access expert medical consulting services, including personalized health advice, answers to daily health-related queries, and predictive health reports. To ensure the precision and reliability of PH-LLM’s data analysis, this study has incorporated multiple high-quality medical resources. These include extensive medical literature, clinical guidelines, health datasets, and insights from clinical experts. Using knowledge extraction techniques, the system synthesizes key medical information such as disease definitions, symptoms, diagnostic criteria, treatment recommendations, and preventive measures.

**Figure 6:**
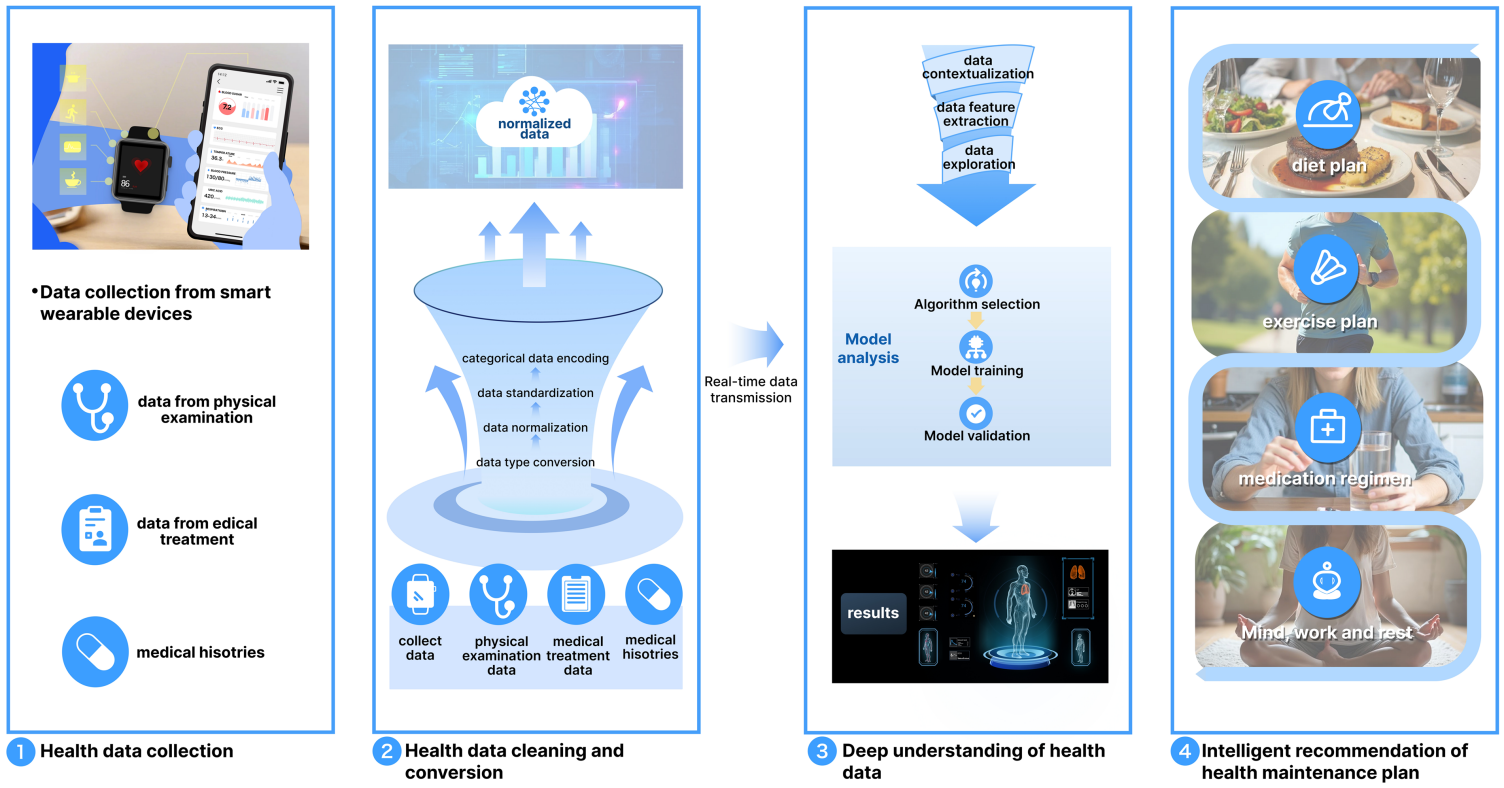
Intelligent recommendation system design for health maintenance solutions.

Against this background, the process of an intelligent system for health maintenance solution involves the following steps: (1) Data Integration. The large language model PH-LLM begins by employing a data integration module to aggregate personal health information, forming a unified database. These data may include noise, missing values, or outliers, and thus undergo cleaning and conversion. Effective cleaning ensures data quality and reliability by employing interpolation methods to estimate missing values and statistical approaches to identify and manage outliers. (2) Deep Understanding of Health Data. After health data are collected, the system performs a “deep understanding” analysis to transform isolated data into meaningful health insights and actionable recommendations. This analysis involves identifying complex patterns and relationships within the data. For instance, a user’s heart rate may have different implications depending on activity levels. A low resting heart rate might indicate relaxation and good health, whereas a high heart rate post-exercise reflects a normal physiological response. The deep understanding module interprets such variations to generate accurate health insights. (3) Intelligent Recommendations for Health Maintenance Plans. The cleaned and analyzed data is then processed through the PH-LLM to generate personalized health recommendations in areas such as diet, exercise, medication, rest, and work. For example, if the smart wearable detects signs of hypertension, the system, powered by PH-LLM, can suggest a tailored plan. This may include dietary adjustments to lower blood pressure, appropriate types of aerobic exercise, optimized sleep schedules, and guidance on medication routines (see Figure 6).

## 5 Conclusions and limitations

Traditional health management models have primarily focused on managing existing diseases. Often, diseases are already in their middle or late stages by the time symptoms are detected. Daily health assessments are frequently overlooked, with little emphasis on preventing potential health issues. In contrast, modern PHM models prioritize not only disease management but also the prevention of future diseases. With the growing demand for healthcare and the increasing popularity of proactive health management, the field of health design is poised for significant development. In recent years, wearable health devices, such as health bracelets and smartwatches, have become widely used. However, the display of physiological values often emphasizes the medical perspective, overlooking the needs of common users who may not fully understand these values or medical data. Furthermore, many smart wearable devices are limited to monitoring physiological indicators, exercise data, and sleep patterns, without offering personalized or intelligent health management services. These devices also fail to address the health anxiety that users experience when confronted with physiological data without clear guidance or context.

To address this issue, it is essential to provide users with clear, easily understandable health information and to guide them in adopting proactive health behaviors. Unlearn and the MILBox research project at the University of Miami’s Miller School of Medicine have already developed a HDT using DT technology. Building on this foundation, the present study further proposes a PHM-HDT model that links DTs with smart wearables. By analyzing the process of creating a DT model and focusing on the characteristics of PHM scenarios, this study develops a PHM-HDT generation model capable of dynamically reflecting the user’s health status. This HDT generation model consists of four layers: the data monitoring layer, data aggregation layer, modeling and computing layer, and human-computer interaction layer. From the perspective of the user’s health experience, this study integrates the HDT as a digital touchpoint throughout the entire process of PHM. It also builds a PHM PSS around three core components: the health visualization and early warning system, the remote collaborative diagnosis and treatment system, and the intelligent recommendation system for health maintenance plans.

In summary, the PHM-PSS proposed in this research maximizes the advantages of the HDT. It enables the visualization of daily health information and supports proactive health management. The system offers strong support for enhancing users’ health management awareness, optimizing their daily health management experience, improving the telemedicine process, and fully leveraging the benefits of digital care.

Although the technology is sufficiently mature, we must acknowledge that the current system remains an ideal scenario, primarily due to limited collaboration among design, AI, medical, and other related disciplines. In addition, further challenges persist, such as the diverse data formats from different sources. In the future, we aim to expand the application of this system to a broader range of health maintenance scenarios. At the same time, we plan to tailor its implementation to local conditions, supported by the combined efforts of various sectors.

## Data Availability

The original contributions presented in the study are included in the article/supplementary material, further inquiries can be directed to the corresponding author.
